# Neglected Myiasis Wound Presenting With Septic Shock: A Case Report

**DOI:** 10.1002/ccr3.72491

**Published:** 2026-04-10

**Authors:** Niranjan Adhikari, Sunil Kumar Das

**Affiliations:** ^1^ Department of General Practice and Emergency Medicine Rasuwa Hospital Dhunche Nepal

**Keywords:** case report, myiasis, neglected wound, sepsis, septic shock

## Abstract

Myiasis is larval infestation of tissues, particularly common in tropical, and subtropical regions, especially in pre‐existing wounds. Poor hygiene, inadequate wound care, and exposed wounds, can predispose to secondary bacterial infection. We report a case of neglected myiasis wound in a laborer from rural Nepal presenting with septic shock, who was managed with intravenous fluids, antibiotics, wound care, and delayed skin grafting, resulting in complete functional recovery.

## Key Clinical Message

Neglected wounds can be infested by insect larvae and can have secondary bacterial infection, which can progress to septic shock. Prompt sepsis management, larval removal, appropriate wound care, and delayed skin grafting can salvage the limb; highlighting the importance of early source control and wound care in resource‐limited settings.

## 1 Introduction

Myiasis, the infestation of live vertebrate by dipterous larvae [1], is a significant health problem in tropical and subtropical regions, particularly in resource‐limited settings [1, 2]. Myiasis primarily affects skin and wounds, it can also involve mucosal surfaces and internal organs [1, 3]. Myiasis has been reported in neglected and exposed traumatic wounds in Nepal, including open fractures [4], and and insect bite wound [5]. Poor hygiene, inadequate wound care, and close vicinity with domestic animals can result in secondary bacterial infection of the myiasis wound [1, 3].

## 2 Case History and Examination

**FIGURE 1:**
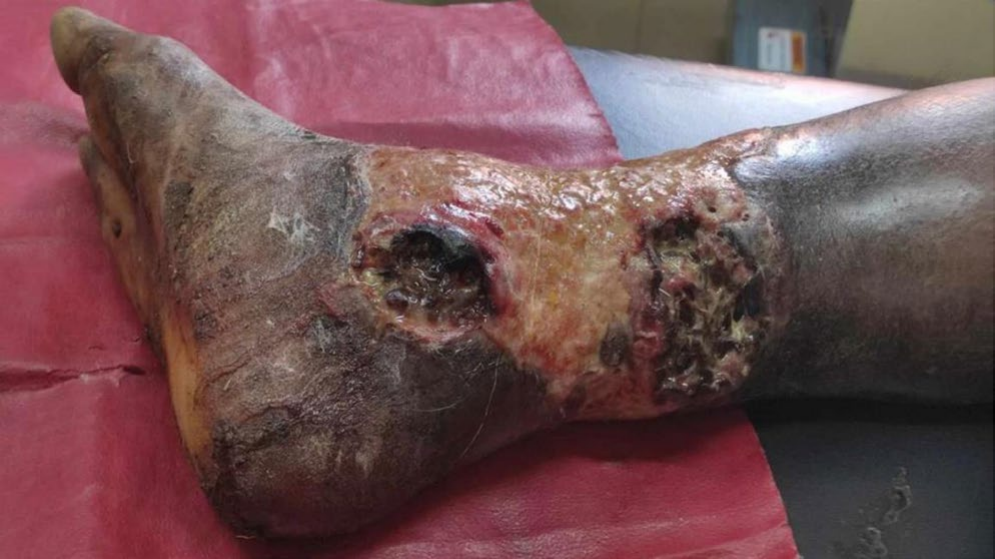
Wound at the time of presentation, infested with maggots, and with necrotic tissues.

A 54 ‐year‐old male with no known comorbidities was brought to the emergency department with a fever for 7 days and a decreased level of consciousness for 3 days. The patient reported that he had a burn injury on his right leg from cooking oil sustained while working in the kitchen 2 months ago. The patient was a daily wage laborer working in India, and could not afford treatment there due to financial constraints. He used to drink alcohol daily to reduce pain, and continued working with wounded leg. The wound progressively increased in size over time. He started having malaise for 2 weeks, followed by a febrile sensation for 7 days. Since he started feeling sleepy all the time and could not go to work, he returned to his village in Nepal. Police officers found him unconscious beside the road and brought him to the emergency department. He used to consume around 500 mL of local alcohol daily for the last 10 years and smoke 5 cigarettes daily for 10 years. At the time of admission, his Glasgow Coma Scale was 11/15 (eye opening: only to pain‐2, best verbal response: confused‐ 4, best motor response: moves to localized pain‐ 5), temperature 101°F, blood pressure 70/40 mmHg, pulse 110/min, and respiratory rate 18/min. Chest and abdominal examination was normal, neurological examination revealed drowsiness and mild confusion. A large, circumferential full thickness wound was observed on his lower right leg, which was infested with maggots, foul‐smelling, with pus, and necrotic tissues. Distal neurovascular status of affected limb was intact (Figure 1).

## 3 Investigations and Treatment

Laboratory investigations revealed a total leukocyte count of 18,000/mm^3^ with 92% neutrophils and 5% lymphocytes, a blood urea level of 90 mg/dL, and a serum creatinine level of 2 mg/dL. A 1000 mL intravenous fluid bolus of Ringer's Lactate was administrated, followed by another 500 mL of Ringer's Lactate in the emergency room. However, his blood pressure was only 76/44 mmHg after 1500 mL fluid administration, following which Noradrenaline infusion was started at 0.1 mcg/kg/min. Tetanus toxoid vaccine was also provided. Empirical antibiotic therapy was initiated with intravenous Ceftriaxone 2 g immediately, then 1 g twice daily for 10 days, followed by oral Cloxacillin for the next 2 weeks. The choice of antibiotic was based on clinical status, and the availability of free‐of‐cost medicine at our hospital. Soft tissue imaging, blood culture, and wound cultures were not done because of the unavailability of these tests at our center, and the inability to refer to a higher center, due to the financial concerns of the patient. He regained full consciousness on 2nd day of admission. On 3rd day of admission, his Mean Arterial Pressure was consistently above 65 mmHg, so Noradrenaline infusion was stopped.

**FIGURE 2:**
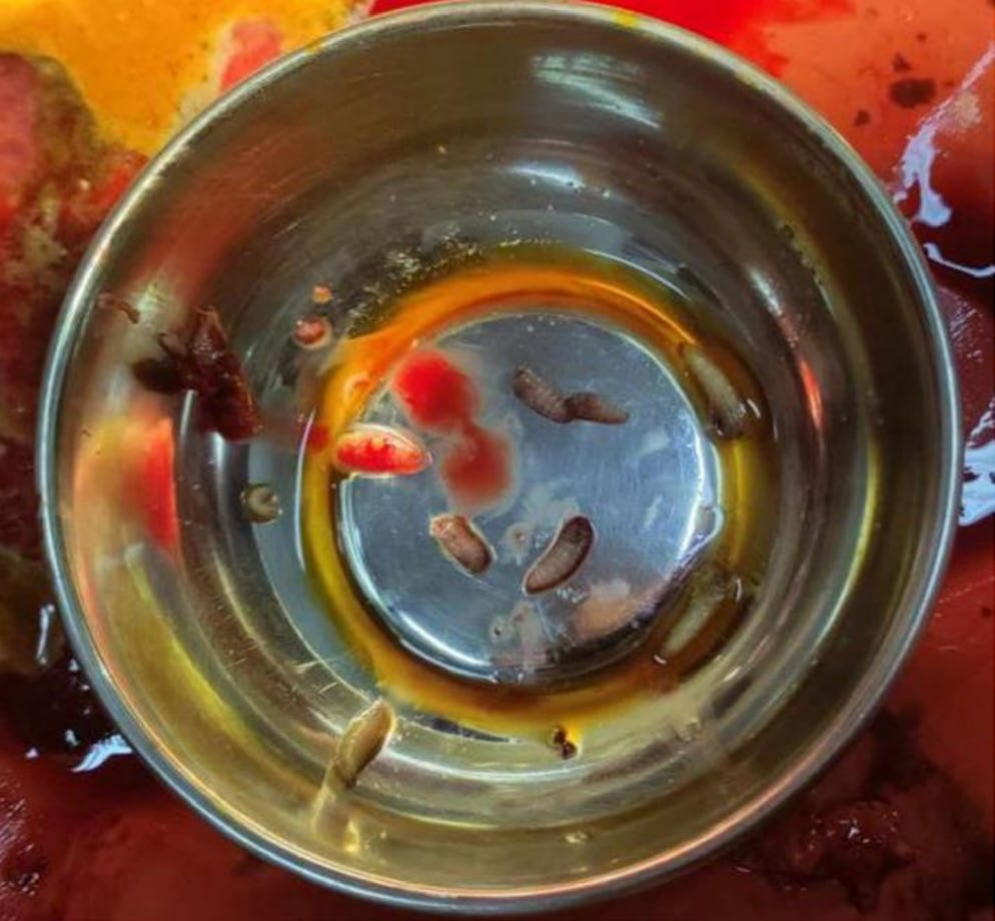
Larvae were extracted from the wound.

**FIGURE 3:**
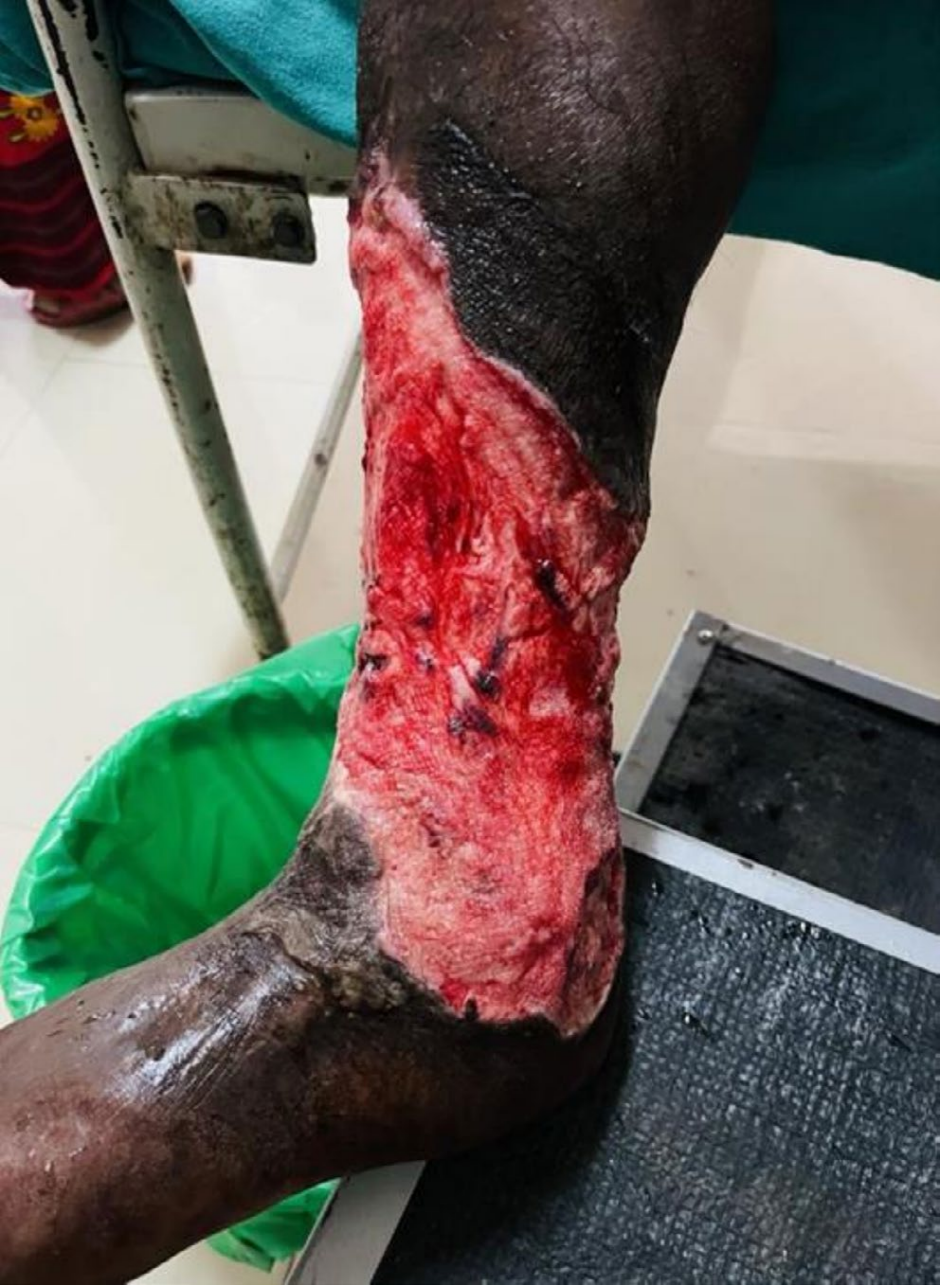
Wound after the extraction of larvae and the debridement of necrotic tissues.

Initially, the wound was irrigated with sterile saline, and maggots were manually removed using forceps and turpentine oil. This procedure was repeated multiple times to ensure complete removal (Figure 2). The wound was initially cleansed with Povidone‐iodine. All necrotic and infected tissue was excised to create a clean wound bed. Hemostasis was achieved using simple ligation and pressure dressings (Figure 3). Wound dressing was done daily with sterile gauze soaked in paraffin and Mupirocin. A healthy wound bed with granulation tissue was achieved only after 3 weeks, following which a split‐thickness skin graft, harvested from the patient's thigh was placed over the wound.

## 4 Outcome and Follow‐Up

**FIGURE 4:**
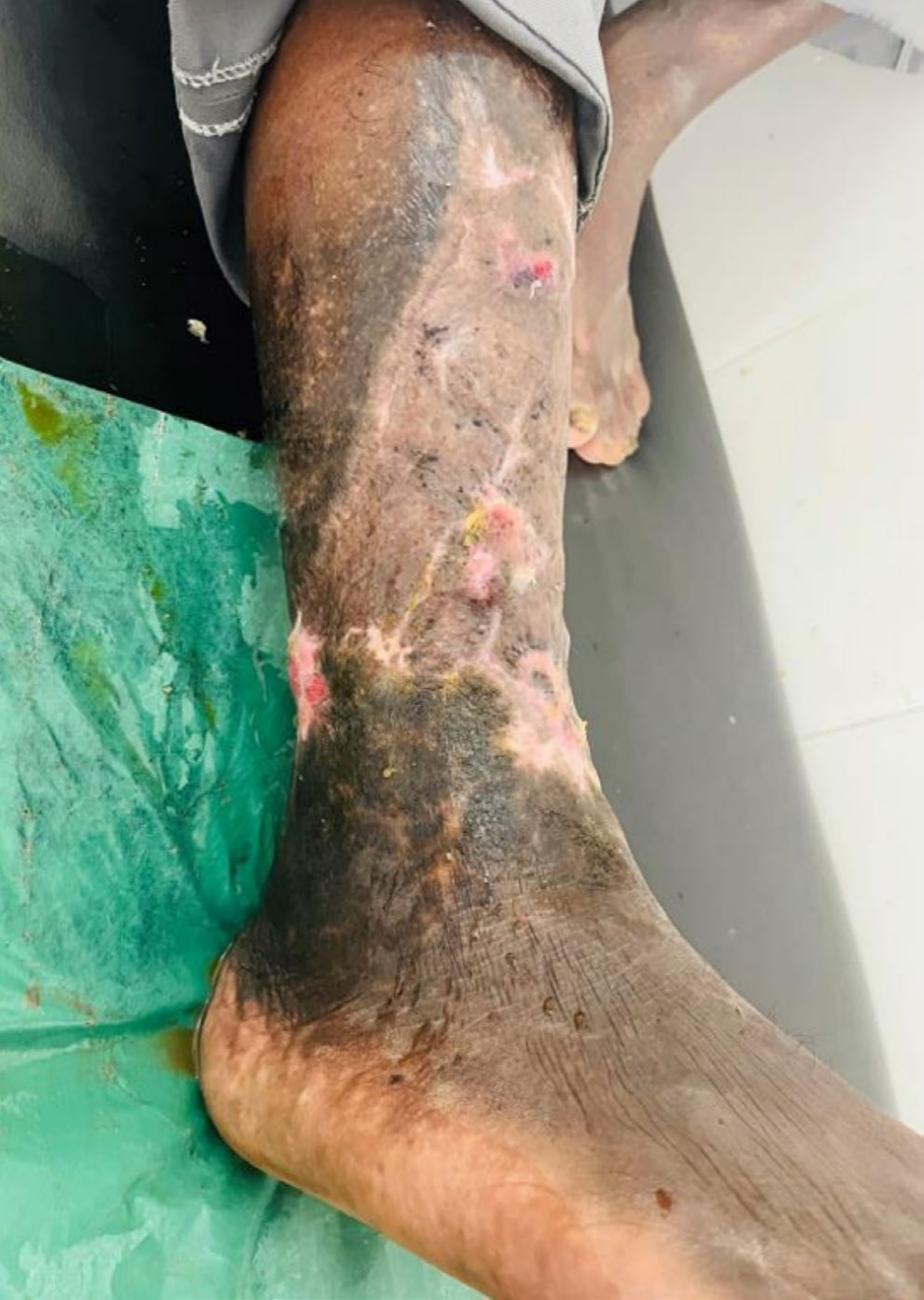
Wound after uptake of skin graft at 3 months follow‐up.

The graft uptake was good, and the patient had full functional recovery. Follow‐up visits after 1 month, 3 months, 6 months, and one year were normal (Table 1). The patient started going to work in his own village after 1 month of discharge from the hospital, and his quality of life improved significantly (Figure 4).

## 5 Discussion

In low‐ and middle‐income countries, inability to afford healthcare expenditures has been identified as a primary barrier in seeking healthcare, with geographical access and availability of healthcare professionals and services being other important barriers [6]. Availability of essential healthcare services in remote settings, utilizing outreach clinics and peripheral healthcare facilities, along with financial subsidies for the poor, is essential for early wound care access and health services in rural areas [6]. In our patient, Ceftriaxone was used empirically, as a broad spectrum antibiotic covering the majority of Gram‐positive as well as negative bacteria, followed by Cloxacillin, considering its coverage against Staphylococci and Streptococci, which are the most common pathogens in burn wound infections and other chronic wound infections [7, 8]. Freshly emerged sterile larvae of certain species of insects, like *Lucilia sericata*, have been used for debridement of chronic infected wounds in controlled clinical settings [9, 10]. However, mistreatment with larvae [11], and infestation of the wound by environmental non‐sterile larvae can worsen the wounds. Secondary bacterial infection in myiasis wounds can have a wide range of presentation, from mild infection to sepsis [12]. If left untreated, it can even present with septic shock, as in our case. With meticulous wound care, control of infection, and skin grafting, complex wound infections can be managed even in resource‐limited settings. Timely recognition, healthcare attention, and appropriate management of wounds is essential to prevent life‐threatening complications like septic shock.

**TABLE 1:** Timeline of events.

2 months before admission: Burn injury on the right leg
2 weeks before admission: Onset of malaise
7 days before admission: Febrile sensation
3 days before admission: Excessive sleepiness and inability to work
Day of admission: Found unconscious beside the road by the police; emergency management including removal of maggots, debridement of wound, antibiotics administration, and initiation of Noradrenaline infusion
Second day of admission: Full consciousness regained
Third day of admission: Noradrenaline infusion stopped
3 weeks after admission: Split‐thickness skin grafting
1 month after admission: Successful graft uptake and full functional recovery
3 months, 6 months, and 12 months after admission: Healed wound; Patient returned to his usual state of health

## 6 Patient Perspective

Patient was grateful for free treatment made available at the hospital, and for being able to go back to work. Also, after discharge from hospital, he started working in his own village in Nepal, living with his family. He also mentioned that he did not know when he would return to his homeland, had he not become ill. For that reason, he once told during the follow‐up visit that the illness occurred for good to him.

## Author Contributions

**Niranjan Adhikari:** conceptualization, writing – original draft, writing – review and editing. **Sunil Kumar Das:** conceptualization, supervision, writing – original draft, writing – review and editing.

## Funding

The authors have nothing to report.

## Disclosure

All the authors declare that the information provided here is accurate to the best of our knowledge.

## Consent

Written informed consent was taken from the patient for the publication of the case report and clinical images.

## Conflicts of Interest

The authors declare no conflicts of interest.

## Data Availability

The data that support the findings of this study are available from the corresponding author upon reasonable request.
